# Molecular Characterization of Small Extracellular Vesicles in Pancreatic Cancer Patients Treated with Neoadjuvant Chemotherapy Followed by Stereotactic Body Radiation

**DOI:** 10.3390/cancers18111704

**Published:** 2026-05-23

**Authors:** Ravi Kumar Paluri, Ashish Kumar, Yixin Su, Sangeeta Singh, Olumide B. Gbolahan, Ashish Manne, Upender Manne, Gagan Deep

**Affiliations:** 1 Department of Cancer Medicine, Atrium Health Wake Forest Baptist Comprehensive Cancer Center, Winston-Salem, NC 27157, USA; ravi.paluri@wfusm.edu; 2 Department of Internal Medicine-Gerontology and Geriatric Medicine, Wake Forest University School of Medicine, Winston-Salem, NC 27157, USA; ashish.kumar@wfusm.edu (A.K.); yixin.su@advocatehealth.org (Y.S.); sangeeta.singh@advocatehealth.org (S.S.); 3 Division of Hematology Oncology, Emory University School of Medicine, Atlanta, GA 30322, USA; ogbolah@emory.edu; 4 Department of Internal Medicine, The Ohio State University, Columbus, OH 43210, USA; ashish.manne@osumc.edu; 5 Department of Pathology, University of Alabama at Birmingham, Birmingham, AL 35233-7331, USA; 6 Division of Hematology Oncology, Comprehensive Cancer Center, University of Alabama at Birmingham, Birmingham, AL 35294-3300, USA; 7 Department of Cancer Medicine, Atrium Health Wake Forest Baptist Comprehensive Cancer Center, Wake Forest University School of Medicine, Winston-Salem, NC 27157, USA

**Keywords:** pancreatic cancer, small extracellular vesicles, neoadjuvant chemotherapy, stereotactic body radiation therapy, liquid biopsy

## Abstract

**Background/Objectives**: Pancreatic ductal adenocarcinoma (PDAC) is a devastating disease with limited therapeutic options, a high mortality rate, and poor overall survival, necessitating the development of new therapeutic and diagnostic strategies. This study investigated the potential of plasma-derived small extracellular vesicles (sEVs) as a source of molecular biomarkers associated with the treatment response. **Methods:** Plasma samples were obtained from patients with locally advanced and borderline resectable PDAC at baseline and following neoadjuvant chemotherapy, either FOLFIRINOX (5-FU [fluorouracil], leucovorin, oxaliplatin, and irinotecan) or GEM-ABRAX ( gemcitabine plus nab-paclitaxel), followed by stereotactic body radiation therapy (SBRT). sEVs were isolated from plasma at baseline, after neoadjuvant chemotherapy, and following SBRT, and were characterized by nanoparticle tracking analysis (NTA), transmission electron microscopy (TEM), nano-flow cytometry, and real-time PCR (RT-PCR). **Results:** The isolated sEVs exhibited an average size of <200 nm, expressed canonical exosome markers (CD63 and CD9), and exhibited pancreatic cancer (PanC)-associated markers, including cholecystokinin A receptor (CCK-AR) and carbohydrate antigen 19-9 (CA19-9). The sEV cargo included several PanC-associated microRNAs (miRNAs). Notably, the expression profiles of these miRNAs demonstrated interpatient variability, though a subset of miRNAs showed statistically significant changes following treatment. **Conclusions:** These findings support the feasibility of sEV isolation and molecular profiling from patient plasma and warrant further investigation as a potential source of biomarkers in pancreatic cancer.

## Simple Summary

Pancreatic ductal adenocarcinoma is an aggressive cancer with limited tools to monitor treatment response in real-time. Blood-based biomarkers that can be serially accessed repeatedly during therapy are needed. In this study, we evaluated small extracellular vesicles isolated from blood samples of patients with locally advanced or borderline resectable pancreatic cancer treated with neoadjuvant chemotherapy followed by stereotactic body radiation therapy. These vesicles were analyzed for pancreatic cancer-associated surface proteins and microRNAs at multiple treatment time points. We observed treatment-related changes in vesicle-associated markers that differed between treatment regimens. Our findings demonstrate that plasma-derived extracellular vesicles can aid in the molecular characterization of tumor response during therapy, supporting their potential use as minimally invasive biomarkers to assess treatment response and disease progression in pancreatic cancer.

## 1. Introduction

Pancreatic ductal adenocarcinoma (PDAC) is a highly lethal malignancy, accounting for more than 90% of all pancreatic cancers, with steadily increasing incidence and mortality rates over the decades [1]. It is currently the third leading cause of cancer-related deaths in the United States, with an overall 5-year survival rate across all stages of about 13%, and for stage IV disease, it is a mere 1–3% [1,2]. These poor outcomes are largely attributed to late diagnosis, aggressive tumor biology, a highly metastatic phenotype, and resistance to conventional therapies [3]. Moreover, the lack of early detection markers and tools results in 35% of patients being diagnosed with locally advanced PDAC and 45% presenting with metastatic disease at diagnosis [4]. The first-line treatment options for PDAC include surgery, chemotherapy, radiotherapy, and/or a combination of these modalities. Although surgical resection remains the primary treatment option, the majority of patients present with unresectable disease due to late diagnosis, an aggressive tumor microenvironment, and a complex, dense stromal architecture characterized by poor vascularization [5]. These features limit effective drug delivery and reduce the efficacy of systemic therapies. More recently, adjuvant chemotherapies like modified FOLFIRINOX (combination of 5-FU [fluorouracil], leucovorin, oxaliplatin, and irinotecan) and gemcitabine plus nab-paclitaxel (GEM-ABRAX) have demonstrated improved survival in randomized clinical trials, with relatively better overall outcomes reported for FOLFIRINOX [6]. However, the optimal selection between these two frontline therapies remains unclear due to the absence of robust biomarkers capable of predicting treatment response or capturing therapy-induced molecular alterations. Therefore, there is an urgent need to improve early diagnostic strategies, predictive biomarkers, and real-time monitoring tools to guide treatment decisions in PDAC.

Currently, serum carbohydrate antigen 19-9 (CA19-9) is widely used in clinical practice. However, it has limited sensitivity and specificity, including false-negative results in individuals with the Lewis-negative phenotype and false-positive results in benign conditions such as cholestasis or inflammation [7]. Although tissue biopsies provide valuable molecular and histopathological insights, they are invasive, prone to sampling bias, and not suitable for longitudinal monitoring. As a result, there is increasing interest in minimally invasive ‘liquid biopsy’ approaches for real-time tumor profiling.

Extracellular vesicles (EVs) have emerged as attractive candidates in this context. EVs are lipid membrane-bound vesicles released by all cell types that mediate intercellular communication by transporting proteins, nucleic acids, metabolites, and lipids that reflect the pathophysiological state of their parent cells, including conditions such as hypoxia, inflammation, and exposure to therapeutic agents [8,9,10,11]. The molecular composition of EV cargo is mostly not random, being regulated by selective sorting mechanisms during vesicle biogenesis. Exosomes, the most-studied subtype of EV, are generated through inward budding of the endosomal membrane to form multivesicular bodies, which, upon fusion with the plasma membrane, release exosomes into the extracellular environment [12,13,14]. Within the heterogeneous EVs, small EVs (sEVs, <200 nm, including exosomes) are of particular interest due to their ability to cross tissue barriers, stability in circulation, and critical role in mediating intercellular communication in cancer progression, metastasis, and treatment response [15,16].

sEVs are well recognized as critical mediators in PDAC, facilitating crosstalk within the tumor microenvironment and distant organs, contributing to processes such as immune evasion, angiogenesis, chemoresistance, stromal reprogramming, and metastasis [17,18]. Importantly, the overwhelming majority of PDAC cases are diagnosed at advanced, unresectable stages due to the limited availability of sensitive and specific tools for early detection [19]. Emerging evidence suggests that specific proteins, mRNA, microRNAs (miRNAs), and DNA fragments encapsulated in sEVs may distinguish early-stage PDAC from benign pancreatic disease and healthy controls, often complementing or, in some contexts, outperforming conventional biomarkers, such as CA19-9 [17,20,21]. The ability to isolate and characterize sEVs from patient plasma, therefore, provides a unique, minimally invasive opportunity to gain insights into the molecular landscape of pancreatic tumors and their response to therapy. Thus, in the current study, we prospectively evaluated patients with locally advanced or borderline resectable PDAC undergoing neoadjuvant chemotherapy followed by stereotactic body radiation therapy (SBRT) (NCT03600623). We isolated and molecularly characterized sEVs from plasma samples collected at multiple time points during treatment to explore their potential as predictive and prognostic biomarkers in this clinical context.

## 2. Materials and Methods

### 2.1. Study Design and Patient Population

Pancreatic cancer patients (*n* = 22) with locally advanced and borderline inoperable disease were recruited at the University of Alabama Comprehensive Cancer Center after IRB approval (clinical trial information: NCT03600623). Patients were administered either FOLFIRINOX or GEM-ABRAX for 2 months, followed by SBRT (33 Gray in 5 fractions). The primary objective of this single-center pilot study was to evaluate the safety and tolerability of this treatment regimen. Further, blood was collected at pre-specified endpoints for feasibility analyses of sEV-based biomarkers.

### 2.2. sEV Isolation

The sEVs were isolated from plasma by a similar ultracentrifugation method as described by us previously [22,23]. Briefly, the plasma samples were first subjected to sequential centrifugation at 500× *g* and 2000× *g* for 5 min at 4 °C to remove cellular debris. The supernatant was then centrifuged at 10,000× *g* for 30 min at 4 °C and then filtered with a 0.22-micron filter to remove larger vesicles. Further, the supernatant was centrifuged at 100,000× *g* for 2 h at 4 °C in a 70.1Ti rotor (Beckman Coulter, Brea, CA, USA). The resulting pellet was resuspended in PBS. Protein concentration was determined by nanodrop (ThermoFisher Scientific, Waltham, MA, USA).

### 2.3. Nanoparticle Tracking Analysis (NTA)

Quantification of the hydrodynamic diameter and concentration of sEVs was estimated by Nanosight NS300 (Malvern Panalytical, Malvern, UK). The instrument was primed with PBS (pH 7.4), and the temperature was maintained at 25 °C. For each sample, five measurements, 30 s each, were taken. The average of five measurements was plotted to represent the size distribution and concentration (particles/mL).

### 2.4. Immunogold Labeling and Transmission Electron Microscopy (TEM)

The characterization of sEVs for the presence of typical exosomal markers on their surface was performed by immunogold labeling using the method described by us previously [24,25]. Briefly, sEVs were fixed with an equal volume of 4% paraformaldehyde for 10 min at room temperature (RT) and then adsorbed for 1 h on 200 mesh copper grids. The grids were washed 3 times (5 min each) with PBS, followed by treatment with 50 mM glycine. After washing, grids were blocked with blocking buffer (0.5% BSA in PBS) for 30 min at RT and then incubated with primary antibodies (1:100) CD63 (ab59479; Abcam, MA, USA), or CD9 (MA5-31980; ThermoFisher, Waltham, MA, USA), overnight at 4 °C. The grids were washed with 0.5% BSA in PBS (5 min × 3) and incubated with secondary antibodies tagged with 10 nm gold particles for 2 h at RT in the dark. The grids were then treated with 2.5% glutaraldehyde for 5 min and incubated with 1% uranyl acetate for 1 min before washing with distilled water for 2 min. A negative control included sEVs treated with gold-labeled secondary antibody only, without the treatment of the primary antibody. Grids were imaged using TEM (FEI Tecnai Spirit transmission electron microscope system, Eugene, OR, USA).

### 2.5. Flow Cytometry

For flow cytometry analysis, sEVs were incubated with fluorescently (AF647) labeled CA19-9 (R&D; FAB10625R) or cholecystokinin A receptor (CCK-AR) (R&D; FAB2680R) antibodies for 2 h at RT in the dark. sEVs were further stained with a membrane labeling dye, MEMGLOW 488 (Cytoskeleton, Inc., Denver, CO, USA, Cat. No. NC1950107), for 15 min at room temperature. All sEV samples were diluted 50-fold in 0.1 μm filtered Tris-EDTA (TE) buffer and analyzed on a nano-flow analyzer (NanoFCM Co., Ltd., Xiamen, China) equipped with 488 nm and 638 nm lasers. The instrument was pre-calibrated with 250 nm silica nanospheres (quality control (QC) beads) and a set of silica nanosphere cocktails of 68, 91, 113, and 155 nm (NanoFCM). The data were acquired under standardized conditions of 10 mW laser power, 10% SS decay, and 1 kPa sampling pressure. Tris-EDTA buffer was run between the samples to clean the instrument and reduce sample cross-contamination.

### 2.6. miRNA Expression Analysis in sEVs

The expression levels of a panel of 11 specific miRNAs (miR-155-5p, miR-106b-5p, miR-16-5p, miR-21-5p, miR-194-5p, miR-181-5p, miR-17-5p, miR-196a-5p, miR-320a-5p, miR-210-3p, miR-509-5p) were analyzed by Taqman-based qPCR assays using the method detailed by us previously [24,26]. Briefly, 400 µL of TRIzol reagent was added to 100 µL of RNase-treated sEVs and incubated for 10 min at RT with frequent vortexing. Next, 100 µL of chloroform was added and mixed by inverting the tube several times before centrifugation at 12,000× *g* for 15 min at 4 °C. The top clear phase was mixed with 3 volumes of 100% ethanol and incubated at −20 °C overnight. The samples were then transferred to the RNase mini spin columns, and total RNA was isolated as per the manufacturer’s protocol (Qiagen, Germantown, MD, USA). The concentration of RNA was measured using NanoDrop (ThermoFisher Scientific, Waltham, MA, USA). For quantitative analysis of miRNA expressions, 25 ng of total RNA was reverse-transcribed into cDNA with the TaqMan Advanced miRNA cDNA Synthesis Kit (A28007, ThermoFisher, Waltham, MA, USA) and amplified for 14 cycles. The cDNA was subsequently diluted 3-fold, and 1 µL of the diluted cDNA was employed for the qPCR analysis using the miRNA-specific TaqMan Advanced miRNA Assay (20×) (ThermoFisher, Waltham, MA, USA) in a 10 µL reaction. Relative expression of miRNAs was normalized with an external control (cel-miR-39-3p) to calculate ΔCt values. Further, the mean value of the control group was used to calculate the ΔΔCt value for all samples. All samples corresponding to a given miRNA were analyzed on the same plate to minimize batch effects, and each sample was analyzed in triplicate for every miRNA. The average of 3 (or more) runs was used to calculate the fold changes.

### 2.7. Statistical Analysis

The preparation of bar graphs and statistical analysis were performed by GraphPad Prism 7 software (GraphPad, San Diego, CA, USA). To signify the comparison between groups, a two-tailed unpaired or paired Student’s *t*-test was used. A *p*-value of <0.05 was considered statistically significant.

## 3. Results

### 3.1. Characterization of sEVs

**Figure 1:**
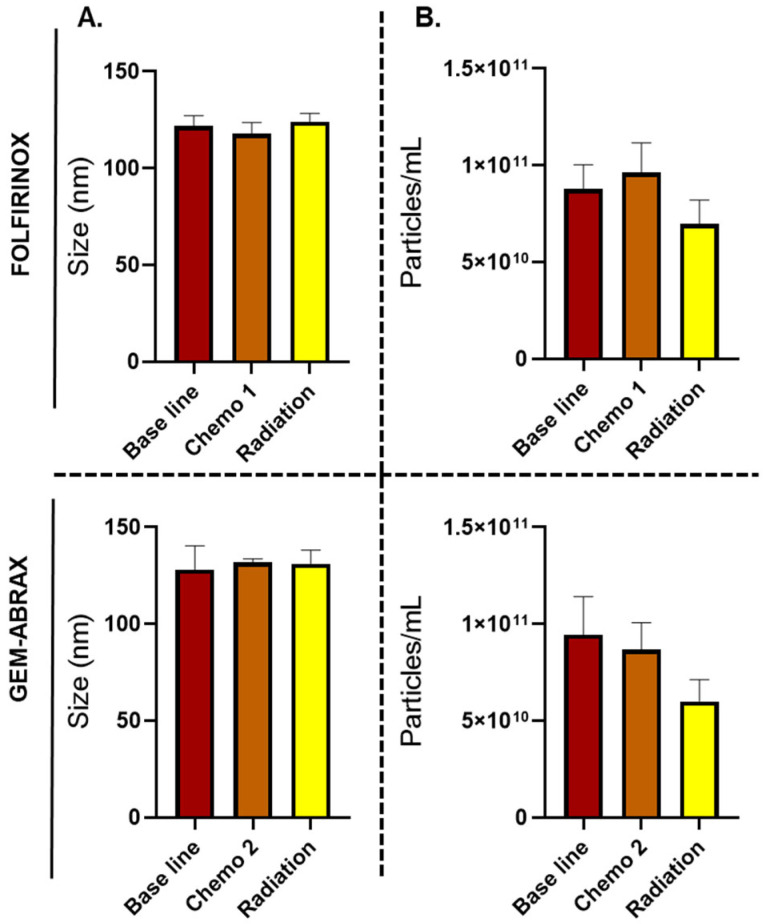
Nanoparticle tracking analysis (NTA) was performed to analyze the size (nm) (**A**) and concentration (particles/mL) (**B**) of total sEVs isolated from the plasma of 2 groups of PDAC patients; group 1 included (i) baseline patients (*n* = 13), (ii) chemotherapy (Chemo 1) followed by FOLFIRINOX (*n* = 10), and (iii) radiation (SBRT) after chemotherapy (*n* = 8); group 2 included (i) baseline (*n* = 9), (ii) chemotherapy (Chemo 2) followed by GEM-ABRAX (*n* = 8), and (iii) radiation (SBRT) after chemotherapy (*n* = 7).

**Figure 2:**
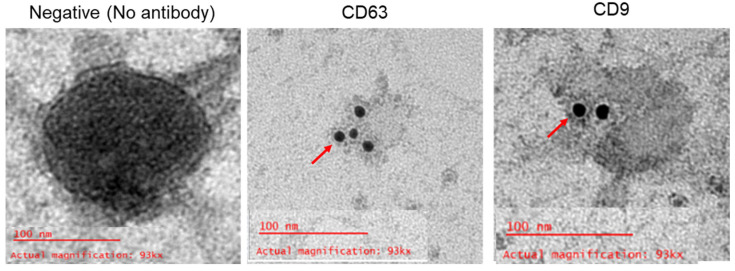
Representative TEM images of sEVs following immuno-gold labeling. Magnification and scale bars are presented at the bottom of each image. Black dots (pointed by red arrows in the image) represent the expression of CD63 or CD9.

Total sEVs isolated from the plasma of all individuals were first analyzed for their size and concentration using NTA. NTA data showed consistent sEVs isolation across treatment groups and represented the size in the sEVs range (<200 nm) (Figure 1A). No significant difference in the mean size was detected among all patients (Figure 1A). We also did not observe any significant changes in the total sEVs concentration, though a marginal reduction was observed following SBRT in both treatment regimens (Figure 1B). Immunogold labeling followed by TEM showed the expression of exosomal markers CD63 and CD9 on the surface of sEVs (Figure 2).

### 3.2. Pancreatic Cancer Biomarkers’ Expression on the Surface of sEVs

**Figure 3:**
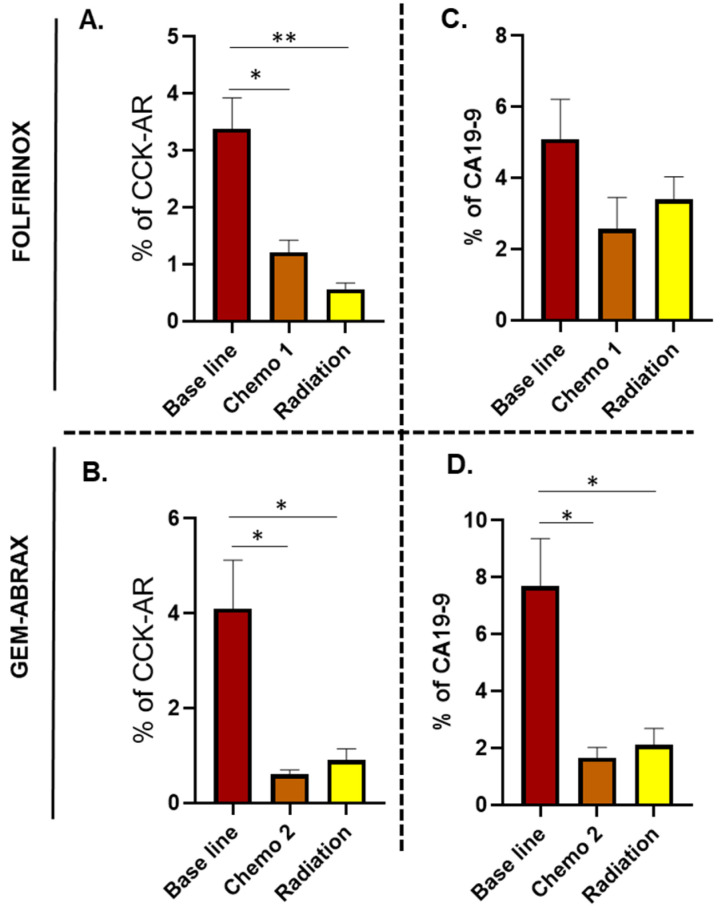
Nano-flow cytometry analysis was used to characterize the surface expression of CCK-AR (**A**,**B**) and CA19-9 (**C**,**D**) on sEVs isolated from the plasma of both groups of pancreatic cancer patients, as shown. Group 1 included (i) baseline patients (*n* = 13), (ii) chemotherapy (Chemo 1) followed by FOLFIRINOX (*n* = 10), and (iii) radiation after chemotherapy (*n* = 8). Group 2 included (i) baseline (*n* = 9), (ii) chemotherapy (Chemo 2) followed by GEM-ABRAX (*n* = 8), and (iii) radiation after chemotherapy (*n* = 7). sEVs were labeled with fluorescently labeled specific antibodies and membrane labeling dye, and events captured as outlined in the methodology. *p* * ≤ 0.05. *p* ** ≤ 0.005.

We next characterized the expression of pancreatic cancer biomarkers (CCK-AR and CA19-9) on the surface of sEVs by nano-flow cytometry. For both regimens (FOLFIRINOX and GEM-ABRAX), we observed a significant decrease in the expression of cholecystokinin A receptor (CCK-AR) following neoadjuvant chemotherapy and radiation (Figure 3A,B). We observed a decreased trend for CA19-9 expression following FOLFIRINOX and SBRT, although this did not reach statistical significance (Figure 3C); however, GEM-ABRAX treatment and SBRT significantly reduced the levels of CA19-9 on sEVs’ surface (Figure 3D).

### 3.3. miRNA Expression in sEVs

**Figure 4:**
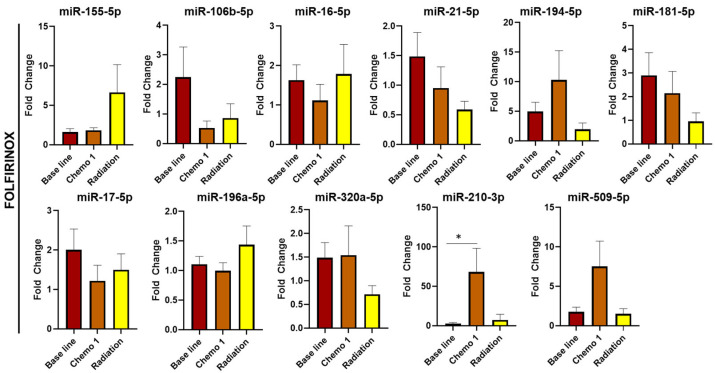
Expression of a panel of miRNAs was analyzed in sEVs isolated from the plasma of patients (as mentioned above) at baseline (*n* = 13), treated with FOLFIRINOX (Chemo 1, *n* = 10), and SBRT (*n* = 8), using Taqman chemistry-based real-time PCR. The change in miRNA expression following chemotherapy and radiation was compared to the baseline patients. The significance of the change in miRNA expression was analyzed using a paired *t*-test. *p* * ≤ 0.05.

**Figure 5:**
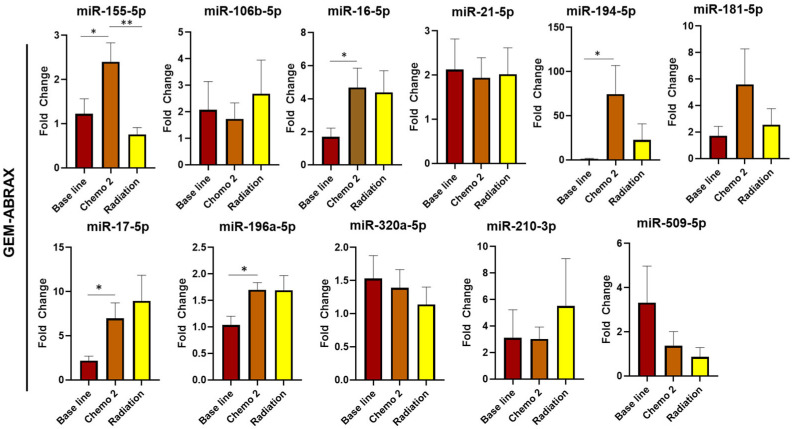
Expression of a panel of miRNAs was analyzed in sEVs isolated from the plasma of patients (as mentioned above) at baseline (*n* = 9), treated with GEM-ABRAX (Chemo 2, *n* = 8) and SBRT (*n* = 7), using Taqman chemistry-based real-time PCR. The change in miRNA expression following chemotherapy and radiation was compared in the baseline patients. The significance of the change in miRNA expression was analyzed using a paired *t*-test. *p* * ≤ 0.05, *p* ** ≤ 0.005.

Next, we analyzed the expression levels of 11 miRNAs (miR-155-5p, miR-106b-5p, miR-16-5p, miR-21-5p, miR-194-5p, miR-181-5p, miR-17-5p, miR-196a-5p, miR-320a-5p, miR-210-3p, miR-50-5p) associated with pancreatic cancer. We observed high variability in the levels of these miRNAs. There was no statistically significant change in any of the miRNAs in the FOLFIRINOX regimen, except for the increased levels of hypoxia-specific miRNA-210-3p, although its levels were reduced after radiotherapy (Figure 4). However, multiple miRNAs showed higher levels following the GEM-ABRAX regimen (Figure 5). We observed a statistically significant increase in the levels of miR-155-5p, miR-16-5p, miR-194-5p, miR-17-5p, and miR-196a-5p following neoadjuvant chemotherapy. Also, the levels of miR-155-5p reduced significantly below baseline after radiation in this group (Figure 5).

## 4. Discussion

This study demonstrates the feasibility of isolating and molecularly characterizing plasma-derived sEVs from patients with PDAC undergoing neoadjuvant chemotherapy followed by SBRT. Using complementary analytical approaches, including NTA, immunogold labeling with TEM, nano-flow cytometry, and miRNA profiling, we show that sEVs can be reliably isolated and retain characteristic size, morphology, and molecular features across treatment time points. These findings establish the technical robustness of our workflow and support the use of sEVs as a platform/tool for longitudinal biomarker assessment in PDAC. We further investigated the characteristics and molecular cargo of plasma-derived sEVs from PDAC patients at baseline, after neoadjuvant chemotherapy with either FOLFIRINOX or GEM-ABRAX, and following SBRT. The clinical trial, upon which this study is based, demonstrated that neoadjuvant FOLFIRINOX or GEM-ABRAX followed by SBRT is feasible, well-tolerated, and associated with antitumor activity in patients with locally advanced pancreatic cancer (LAPC) [27]. This treatment regimen provides valuable clinical context for investigating potential circulating biomarkers that predict real-time treatment response and survival.

The nano-flow cytometry analysis showed treatment (neoadjuvant chemotherapy followed by SBRT)-associated changes in sEV surface expression of PDAC-associated markers, including CCK-AR and CA-19-9. Although CA19-9 remains the only FDA-approved blood-based biomarker for PDAC, its clinical utility is limited by variability in sensitivity and specificity, as well as its increased levels in non-malignant conditions (false-positives) [28,29]. The serum level of CA19-9 in PDAC patients is shown to be associated with the survival of patients with advanced pancreatic cancer [30,31,32]. Importantly, CA19-9 levels after neoadjuvant chemotherapy and surgical resection correlate with the histopathological response and survival rates [33]. In this study, we observed a reduction in CA19-9 levels in the sEVs in both neoadjuvant chemotherapy cohorts, with a statistically significant decrease in the GEM-ABRAX plus SBRT treatment group. Notably, sEV-associated CA19-9 could offer an advantage over serum measurements by reflecting tumor-derived secretion and improving molecular specificity. Moreover, this may potentially circumvent some of the sensitivity limitations of serum-based CA19-9, as vesicle encapsulation may enhance stability. We also identified robust expression of CCK-AR on sEVs, a receptor implicated in PDAC growth and progression through cholecystokinin-mediated signaling pathways [34]. Since CCK-AR is expressed selectively in PDAC [35], its detection in sEVs demonstrates a potentially novel tumor-specific biomarker. Importantly, the observed decrease in the number of sEVs positive for CA19-9 and CCK-AR following neoadjuvant chemotherapy and radiation supports their potential utility as a marker to monitor treatment response and tumor burden.

Previous studies have reported dynamic changes in the levels of key miRNAs associated with pancreatic cancer [36,37,38]. In this study, in addition to protein markers, we observed dynamic and regimen-specific changes in sEV-associated miRNA profiles. Following FOLFIRINOX treatment, miR-210-3p, a hypoxia-specific miRNA, was significantly elevated compared to baseline samples, potentially reflecting therapy-induced tumor hypoxia or microenvironmental stress. This observation is consistent with prior studies linking upregulated expression of miR-210-3p to hypoxia and PDAC progression [39,40]. Additionally, in vitro experiments on pancreatic cancer cells showed that the overexpression of miR-210 leads to cell cycle arrest and reduced cell viability [41]. Thus, the decreased levels of miR-210-3p following SBRT suggest a possible modulation of hypoxic stress after radiation therapy.

In contrast, we observed prominently higher levels of several miRNAs, including miR-155-5p, miR-16-5p, miR-194-5p, miR-17-5p, and miR-196a-5p, following GEM-ABRAX treatment compared to baseline. These miRNAs have previously been linked to PDAC growth, metastasis, and poor clinical outcomes [42,43,44,45,46,47]. For example, miR-155 has been implicated in lymphatic metastasis of pancreatic cancer and suggested as a potential biomarker for detecting the early stage of pancreatic cancer [44,45]. We observed significantly higher levels of miR-155 following GEM-ABRAX treatment and SBRT, possibly reflecting a stress response from cancer cells. Previously, expression profiles of miR-16, miR-196a, and CA19-9 were analyzed in the plasma of 140 pancreatic cancer patients, 111 chronic pancreatitis patients, and 68 normal controls. Importantly, the results demonstrated that the combination of miR-16 and miR-196a with CA19-9 was more accurate in discriminating pancreatic cancer from normal tissue, with a sensitivity and specificity of 92.0% and 95.6%, respectively [47]. Additionally, increased levels of miR-196a and miR-155, along with other miRNAs, are suggested to be associated with a poor survival rate [46]. Together, dynamic changes in the miRNA expression in sEVs across distinct treatment regimens suggest their potential as real-time indicators of therapeutic efficacy. Moreover, the distinct signature of miRNAs in both regimens may reflect tumor cell stress response, selective pharmacological mechanisms, and evolving tumor biology under therapeutic stress.

There are several limitations to this study. Although the sample size was sufficient for exploratory analyses and to demonstrate the feasibility of using plasma sEVs as molecular surrogates for PDAC, it remains modest. Accordingly, validation in larger, independent cohorts using more rigorous study designs will be required to establish robust and clinically meaningful PDAC biomarkers. Additionally, further research is needed to elucidate the precise mechanisms by which sEVs and their molecular cargo contribute to PDAC progression and treatment response. Correlative analyses with tumor tissues were not performed, limiting our ability to directly link circulating sEV profiles with intratumoral molecular changes. Despite these limitations, our study provides important proof-of-concept evidence supporting the feasibility and translational potential of sEV-based liquid biopsy biomarker approaches in PDAC. Serial profiling of sEVs offers a minimally invasive strategy for monitoring treatment response, assessing tumor progression, and potentially guiding personalized therapeutic decisions. Future studies should focus on validating these biomarkers in larger cohorts and elucidating the functional roles of specific sEV-associated molecules in pancreatic cancer progression and treatment resistance.

## 5. Conclusions

In conclusion, this study demonstrates that plasma-derived sEVs can be reliably isolated and longitudinally profiled in patients with PDAC undergoing neoadjuvant therapy, capturing both protein and miRNA changes associated with treatment- response. The findings, particularly related to reduced levels of sEV-associated CA19-9 and CCK-AR, together with regimen-specific miRNA signatures, highlight the potential of sEVs as minimally invasive biomarkers for monitoring therapeutic response. Although these findings require validation in larger cohorts, they form a strong basis for integrating sEV-based analyses into clinical workflows aimed at improving real-time disease monitoring and guiding treatment decisions in pancreatic cancer.

## Data Availability

The datasets generated and analyzed during the current study are available from the corresponding authors upon reasonable request.
